# Cesarean delivery and overweight at 6 years of age: a prospective cohort analysis of the Japan Environment and Children’s Study

**DOI:** 10.1007/s00431-026-07453-w

**Published:** 2026-10-05

**Authors:** Shintaro Terashita, Akiko Tsuchida, Hidekuni Inadera, Chihaya Imai, Eiji Yoshioka, the Japan Environment and Children’s Study Group, Tetsuo Nomiyama, Shin Yamazaki, Maki Fukami, Reiko Kishi, Chiharu Ota, Koichi Hashimoto, Kenichi Sakurai, Shuichi Ito, Sanae Otawa, Michihiro Kamijima, Eiji Yoshioka, Takeo Nakayama, Ryo Kawasaki, Yasuhiro Takeshima, Akemi Morita, Narufumi Suganuma, Mayumi Tsuji, Kimitoshi Nakamura

**Affiliations:** 1 Toyama Regional Centre for Japan Environment and Children’s Study, University of Toyama, 2630 Sugitani, Toyama, Toyama 930-0194 Japan; 2 Department of Pediatrics, Faculty of Medicine, University of Toyama, Toyama, Japan; 3 Department of Public Health, Faculty of Medicine, University of Toyama, Toyama, Japan

**Keywords:** Cesarean delivery, Childhood overweight, Adiposity rebound, Birth cohort, Epidemiology

## Abstract

Cesarean delivery (CD) has been suggested as a potential risk factor for childhood overweight and obesity; however, evidence beyond early childhood remains inconsistent. We examined the association between CD and childhood overweight at age 6 years and explored early life body mass index patterns including adiposity rebound (AR), in a large Japanese birth cohort. We analyzed 64,281 singleton mother-child pairs from the Japan Environment and Children’s Study. Overweight at age 6 years was defined according to the International Obesity Task Force criteria. Logistic regression models were used to estimate adjusted odds ratios (aORs). Changes in overweight status between ages 3 and 6 years were examined to distinguish incident overweight from persistent overweight, and AR timing was evaluated. CD accounted for 18.5% of the births. Overweight at age 6 years was more frequent among children born by CD than among those born by vaginal delivery (10.7% vs. 8.8%). After adjusting for the prespecified covariates, CD was associated with higher odds of overweight at age 6 years (aOR 1.09, 95% confidence interval 1.02–1.16). In sex-stratified analyses, CD was associated with incident overweight between ages 3 and 6 years among females but not among males; however, the interaction between delivery mode and sex was not statistically significant. CD was not significantly associated with earlier AR.

*Conclusion*: In this large Japanese cohort, CD was modestly associated with childhood overweight at age 6 years.

**Supplementary Information:**

The online version contains supplementary material available at https://doi.org/10.1007/s00431-026-07453-w.

**Table:** 

**What is Known:** • *Cesarean delivery (CD) is associated with a moderately increased risk for childhood overweight.* • *Evidence regarding the association between CD and overweight beyond early childhood is limited.*	
**What is New:** • *In this large Japanese birth cohort, CD was associated with overweight in children aged 6 years.* • *CD was associated with a higher incidence of new-onset overweight between ages 3 and 6 years only among girls.*	

## Introduction

Childhood obesity and overweight continue to increase globally and are a major public health challenge. Global epidemiological analyses have indicated that the prevalence of childhood obesity increased approximately 1.6-fold between 1990 and 2010, with a sustained upward trajectory observed across many countries and regions thereafter [1, 2]. In Japan, childhood obesity peaked in the early 2000 s, although it has shown a renewed increase during the past decade [3].

Childhood obesity is not a transient condition; it shapes lifelong health risks. The adipocyte number increases predominantly during childhood and does not substantially decline thereafter [4], and adipose tissue may retain an “epigenetic memory” of obesity, contributing to its persistence [5]. Consequently, childhood obesity increases the risk of adult obesity [6] and early cardiometabolic abnormalities [7–9]. Despite evidence that remission improves long-term outcomes [10], effective population-level prevention strategies are lacking [11], underscoring the need to identify early life determinants.

The development of childhood obesity is a multifactorial process. In addition to lifestyle factors such as excessive caloric intake and physical inactivity, perinatal and early life exposures play important roles. A high maternal pre-pregnancy body mass index (BMI), excessive gestational weight gain, maternal smoking, birth weight, and infant feeding practices have all been reported as risk factors for childhood obesity [12–14]. In addition, cesarean delivery (CD) has recently attracted attention as a potential risk factor [15].

Systematic reviews and meta-analyses have reported that children born by CD have an approximate 15–30% higher risk for obesity in childhood compared with those born by vaginal delivery (VD) [16, 17]. However, establishing a causal relationship remains controversial. Heterogeneity in outcomes, age, differences in adjustment factors, racial and ethnic variations, and differences in follow-up duration have been cited as potential sources of inconsistency. Consequently, the extent to which a single perinatal factor, the mode of delivery, contributes to obesity development beyond early childhood remains unclear.

In our previous study, we used data from the Japan Environment and Children’s Study (JECS), a large nationwide birth cohort in Japan, and demonstrated that CD was significantly associated with an increased risk of childhood obesity at age 3 years [18]. However, whether this association persists into school age or attenuates with growth remains unknown. The age of 6 years represents a critical transition from early childhood to school age and provides an important time point to evaluate whether obesity is transient or tracks into later life [6, 14].

BMI trajectories in early life, particularly adiposity rebound (AR), have emerged as important predictors of obesity and cardiometabolic risk later in life. AR refers to the age at which BMI begins to increase again after declining in infancy, which typically occurs between 6 and 8 years of age [19, 20]. Earlier timing of AR has been associated with an increased risk of adult obesity, insulin resistance, and type 2 diabetes [21–23]. However, only a few studies have examined the relationship between CD and AR.

Therefore, given these considerations, in this study we investigated whether CD is associated with childhood overweight at age 6 years using the large-scale prospective cohort data from the JECS. Additionally, we examined changes in overweight status between 3 and 6 years of age to distinguish newly developed overweight from persistent overweight and evaluated AR timing.

## Methods

### Study design and participants

The present study was based on data obtained from JECS, an ongoing nationwide prospective birth cohort study. The protocol details have been published previously [24, 25]. A total of 104,043 fetal records were registered at 15 Regional Centers across Japan between January 2011 and March 2014. This study used the datasets, which contain prospectively collected information about mothers and their children up to age 6 years. We restricted the present study to singleton children because early growth differs between multiples and singletons [26]. Duplicate registrations were identified using the maternal identification number, and the first registration was retained. The 6-year assessment corresponded to an age range of 5 years 7 months to 6 years 5 months; exact measurement dates were unavailable. BMI values outside ± 5 SDs from the mean were excluded, whereas no additional exclusion criteria were applied to height or weight, gestational age, or congenital anomalies. For the BMI trajectory analysis, participants with BMI data at four or more time points were included.

From the initial cohort, we excluded 5,690 duplicate registrations, 1,910 multiple births, and 3,676 miscarriages or stillbirths to identify mother-child pairs. Among the remaining 92,767 mothers, an additional 247 were excluded because of missing information for delivery mode, 26,189 because of missing or outlier anthropometric data for their children at age 6 years, and 2,050 because of incomplete covariate data. Consequently, 64,281 mother-child pairs were available for complete-case analysis (Supplementary Fig. 1).

### Exposure

Information about the delivery mode was obtained from medical record transcripts completed by physicians, midwives/nurses, and/or Research Co-ordinators.

### Outcomes

#### Primary outcome

The primary outcome was childhood overweight at age 6 years. Overweight was defined according to the age- and sex-specific BMI cut-offs (males 17.55, females 17.34) proposed by the International Obesity Task Force (IOTF) [27]. In sensitivity analyses, IOTF obesity BMI cut-offs (males 19.78, females 19.65) and Japanese-specific BMI cut-offs for overweight (85th percentile: males 17.47, females 17.38) provided by The Japanese Society for Pediatric Endocrinology were also applied [28]. The BMI at 6 years was calculated using height and weight reported by caregivers via questionnaires distributed and collected by mail. To minimize measurement errors, the caregivers were asked to report the most recent height and weight measurements obtained during well-child visits or at medical institutions, and to specify the measurement date. If such data were unavailable, the height and weight measured at nursery school or home were accepted.

#### Changes in overweight status between ages 3 and 6 years

We also examined changes in childhood overweight status from ages 3 to 6 years. The participants were classified according to the presence or absence of overweight at both ages 3 and 6 years based on the corresponding IOTF BMI cut-offs (males 17.89, females 17.56 at 3 years; males 17.55, females 17.34 at 6 years). Four categories were defined according to overweight transition patterns: (1) without overweight at 3 years → without overweight at 6 years (never with overweight), (2) overweight at 3 years → without overweight at 6 years (with remitted overweight), (3) without overweight at 3 years → overweight at 6 years (with incident overweight), and (4) overweight at 3 years → overweight at 6 years (with persistent overweight). The distribution of the participants across these categories was analyzed to evaluate the longitudinal changes in overweight status. For the incident and persistent overweight analyses, the analyses were restricted to children without and with overweight at age 3 years, respectively.

#### BMI trajectory and AR

We investigated the BMI trajectories with a specific focus on AR timing. In this study, AR was defined as the nadir point in BMI occurring after age 2 years that met the following criteria: (1) BMI increased for at least two consecutive measurement points after the nadir, and (2) the increase from the nadir was ≥ 0.3 kg/m^2^, consistent with previous epidemiological studies [29]. When multiple candidate nadir points satisfied these criteria, the latest point was adopted for AR onset. Early AR was defined as AR onset at age ≤ 5 years. To evaluate post-AR weight gain, the BMI slope was calculated as the change in BMI from AR onset to age 6 years divided by the time interval between AR onset and age 6 years (kg/m^2^/year).

### Covariates

Covariates for the primary multivariable analysis were selected using a directed acyclic graph (DAG) [30], constructed a priori based on temporal ordering, clinical plausibility, and previous epidemiological evidence [31–33]. The DAG was used to represent the assumed causal relationships between CD, childhood overweight, and potential confounding or mediating variables. Based on the DAG, maternal pre-pregnancy BMI, maternal smoking, and maternal diabetes mellitus were included as covariates in the primary adjusted model. Potential mediators, including birth size and neonatal complications, were not included in the primary adjustment set. Pregnancy complications were also excluded from the primary model because they may partly determine the mode of delivery. Details of maternal comorbidities, pregnancy complications, and neonatal complications are provided in Supplementary Table 1. The DAG is also provided in Supplementary Fig. 2.

As a sensitivity analysis, CD was classified as elective or emergency based on recorded obstetric indications, as the questionnaire did not directly distinguish between them. Elective CD included repeat CD, history of uterine surgery, placenta previa, and fetal malpresentation; all other specified indications were classified as emergency, with emergency classification prioritized when both types of indications were present [34]. Cases of CD without sufficient information for classification as elective or emergency were excluded from this sensitivity analysis.

### Statistical analyses

Normality of continuous variables was assessed using the Shapiro–Wilk test. Continuous variables that were not normally distributed are presented as medians with interquartile ranges, whereas categorical variables are presented as frequencies and percentages. Differences in BMI between the CD and VD groups were compared using the *t*-test.

For the primary analysis, logistic regression models were used to estimate crude odds ratios (cORs), adjusted odds ratios (aORs), and their 95% confidence intervals (CIs) for the association between mode of delivery and overweight at age 6 years. VD was used as the reference category. The adjusted model included maternal pre-pregnancy BMI, maternal smoking, and maternal diabetes mellitus, as identified using the DAG. Maternal pre-pregnancy BMI of 18.5– < 25 kg/m^2^, non-smoking, and absence of maternal diabetes mellitus were used as the reference categories. Sex-stratified analyses were also performed, and the interaction between delivery mode and sex was assessed by including a multiplicative interaction term.

The same logistic regression models and adjustment set were used to examine incident overweight among children without overweight at age 3 years, persistent overweight among children with overweight at age 3 years, and early AR. Model fit for the primary analysis was evaluated using the Hosmer–Lemeshow goodness-of-fit test and influence diagnostics, including standardized residuals and Cook’s distance. Multicollinearity was assessed using generalized variance inflation factors.

Sensitivity analyses were performed using the IOTF obesity cut-offs, Japanese-specific overweight cut-offs, and the classification of CD as elective or emergency. In addition, mixed-effects logistic regression models with the 15 regional centers included as a random intercept were fitted to account for potential clustering by study region. All logistic regression analyses were conducted using a complete-case approach.

For longitudinal BMI trajectories, mean BMI values and corresponding 95% CIs at each age were calculated using all available observations at each time point. Differences in BMI between the CD and VD groups at each age were smoothed using linear regression with natural spline functions to visualize nonlinear changes over age. These analyses were descriptive.

Linear regression models were used to examine the association between CD and post-AR BMI slope. The adjusted models included maternal pre-pregnancy BMI, maternal smoking, and maternal diabetes mellitus. Regression coefficients (β) and their 95% CIs were estimated.

All statistical tests were two-sided, and *P* < 0.05 was considered statistically significant. All analyses were performed using R software (version 4.5.2; R Foundation for Statistical Computing, Vienna, Austria).

## Results

### Higher childhood overweight prevalence at age 6 years in the CD group

Of the 64,281 mother-child pairs analyzed, 11,871 (18.5%) had a CD birth (Table 1). The proportion of mothers aged ≥ 35 years during pregnancy was higher in the CD group than in the VD group (36.8% vs 23.6%), as was the proportion with a BMI ≥ 25 (15.2% vs 8.1%). The mothers in the CD birth group experienced more maternal and pregnancy complications. There was little difference in highest education level, annual household income, smoking history, alcohol consumption, maternal diabetes, and parity between the CD and VD groups (Table 1). Among the analyzed children, 32,951 (51.3%) were males, and those in the CD birth group had lower median birth height and weight. Baseline characteristics of included and excluded participants are shown in Supplementary Table 2. CD was less frequent among included participants (18.5% vs. 19.9%; SMD = 0.037), and differences were also observed in several maternal and socioeconomic characteristics.

**Table 1:** Characteristics of the study population

Variable		Vaginal delivery *n* = 52,410 *n *(%)	Cesarean delivery *n* = 11,871 *n *(%)
Maternal age (years)	< 25	5,057 (9.6)	706 (5.9)
	25– < 30	16,022 (30.6)	2,573 (21.7)
	30– < 35	18,939 (36.1)	4,221 (35.6)
	≥ 35	12,392 (23.6)	4,371 (36.8)
Maternal BMI (kg/m^2^)	< 18.5	8,997 (17.2)	1,536 (12.9)
	18.5– < 25	39,160 (74.7)	8,529 (71.8)
	≥ 25	4,253 (8.1)	1,806 (15.2)
Parity	Primipara	24,125 (46.3)	5,188 (44.0)
	Multipara	27,982 (53.7)	6,605 (56.0)
Highest education level, years of education	< 16	39,210 (75.4)	9,066 (77.2)
	≥ 16	12,799 (24.6)	2,680 (22.8)
Annual household income, million JPY	< 4	18,513 (37.7)	4,089 (36.9)
	4– < 6	16,675 (34.0)	3,688 (33.2)
	≥ 6	13,878 (28.3)	3,319 (29.9)
Maternal smoking	Never	32,742 (62.5)	7,133 (60.1)
	Former	17,975 (34.3)	4,331 (36.5)
	Current	1,693 (3.2)	407 (3.4)
Maternal alcohol intake	Never	19,001 (36.3)	4,228 (35.7)
	Former	28,517 (54.5)	6,497 (54.8)
	Current	4,790 (9.2)	1,128 (9.5)
Maternal comorbidities	No	44,219 (85.9)	9,296 (79.8)
	Yes	7,272 (14.1)	2,347 (20.2)
Maternal diabetes	No	51,875 (99.0)	11,725 (98.8)
	Yes	535 (1.0)	146 (1.2)
Pregnancy complications	No	50,242 (96.3)	10,841 (91.8)
	Yes	1,951 (3.7)	963 (8.2)
Child sex	Male	26,854 (51.2)	6,097 (51.4)
	Female	25,556 (48.8)	5,774 (48.6)
Neonatal complications	No	47,021 (91.9)	9,369 (81.0)
	Yes	4,132 (8.1)	2,195 (19.0)
Birth height (cm)	Median [IQR]	49.2 [48.0–50.5]	48.1 [46.8–50.0]
Birth weight (g)	Median [IQR]	3,054 [2818–3300]	2,894 [2630–3172]

The analytic sample was restricted to participants with complete data on maternal pre-pregnancy BMI, smoking, and maternal diabetes mellitus. Numbers for other variables may not sum to the total sample size because of missing data. ≥ 16 years of education corresponds to university graduation or higher. JPY 1 million was approximately equivalent to EUR 5,556 (JPY 180/EUR, [September, 2026])

*BMI,* body mass index; *JPY,* Japanese yen; *IQR,* interquartile range

Overall, 5,876 (9.1%) of the children had overweight at age 6 years. Overweight was observed in 1,268 (10.7%) children born by CD and 4,608 (8.8%) children born by VD. Analysis revealed that childhood overweight prevalence at age 6 years was higher among children born by CD than among those born by VD after adjustment for the prespecified covariates (aOR 1.09, 95% CI 1.02–1.16). In the sex-stratified analyses, the adjusted odds were higher among females born by CD (aOR 1.11, 95% CI 1.01–1.22), but not among males (aOR 1.06, 95% CI 0.96–1.17). These results are summarized in Table 2.

**Table 2:** Prevalence of overweight at age 6 years and its association with CD

		N	Overweight at age 6 years *n *(%)	Crude OR (95% CI)	Adjusted OR (95% CI)
Overall		64,281	5,876 (9.1)	—	—
Delivery mode					
Vaginal delivery		52,410	4,608 (8.8)	Ref	Ref
Cesarean delivery		11,871	1,268 (10.7)	1.24 (1.16–1.32)	1.09 (1.02–1.16)
Sex and delivery mode					
Male	Vaginal	26,854	2,176 (8.1)	Ref	Ref
	Cesarean	6,097	588 (9.6)	1.21 (1.10–1.33)	1.06 (0.96–1.17)
Female	Vaginal	25,556	2,432 (9.5)	Ref	Ref
	Cesarean	5,774	680 (11.8)	1.27 (1.16–1.39)	1.11 (1.01–1.22)

Covariates: maternal pre-pregnancy BMI, maternal smoking, and maternal diabetes mellitus were included as covariates. *OR*, odds ratio; *CI,* confidence interval; *Ref,* reference

Similar findings were observed when the IOTF obesity BMI cut-offs were applied. Overall, 1,296 (2.0%) children were classified as having obesity at age 6 years. Although the odds of obesity tended to be higher among children born by CD, the associations did not reach statistical significance overall (aOR 1.13, 95% CI 1.00–1.29), among boys (aOR 1.09, 95% CI 0.91–1.31), or among girls (aOR 1.18, 95% CI 0.97–1.42). Similar results were obtained using the Japanese BMI criteria, under which 5,941 (9.2%) children were classified as having overweight. The aORs were 1.08 (95% CI 1.01–1.16) overall, 1.07 (95% CI 0.98–1.18) among boys, and 1.09 (95% CI 1.00–1.20) among girls.

No significant interaction was observed between delivery mode and sex (P for interaction = 0.50). The Hosmer–Lemeshow test did not indicate poor model fit (*P* = 0.81), and no problematic multicollinearity was observed (adjusted GVIFs, 1.00–1.01).

In the sensitivity analysis separating elective and emergency CD, elective CD was associated with higher odds of overweight compared with VD (aOR 1.12, 95% CI 1.03–1.22), whereas emergency CD was not (aOR 1.05, 95% CI 0.95–1.15). In sex-stratified analyses, the association with elective CD was observed only among girls (aOR 1.15, 95% CI 1.02–1.29).

In addition, mixed-effects logistic regression with the 15 regional centers as a random intercept yielded results consistent with the primary analysis (overall: aOR, 1.09; 95% CI, 1.02–1.17; *P* = 0.01; boys: aOR, 1.07; 95% CI, 0.97–1.18; *P* = 0.18; girls: aOR, 1.12; 95% CI, 1.02–1.23; *P* = 0.02).

### Changes in overweight status from ages 3 to 6 years

We examined the distribution of overweight status at age 6 years according to overweight classification at age 3 years. The children were categorized as with or without overweight at age 3 years based on the IOTF overweight cut-offs, and transitions between ages 3 and 6 years were evaluated.

In the CD group, the proportion of children classified as never having overweight was lower than that in the VD group. In contrast, both incident and persistent overweight were observed more frequently among children born by CD for both sexes.

We calculated the odds ratios for incident overweight among children without overweight at age 3 years and for persistent overweight among children with overweight at age 3 years. After adjustment for potential confounders, the association with incident overweight remained statistically significant among females (aOR 1.14, 95% CI 1.01–1.29), but not among males (aOR 1.08, 95% CI 0.95–1.21). The associations with persistent overweight were not statistically significant among either males (aOR 1.08, 95% CI 0.86–1.36) or females (aOR 1.04, 95% CI 0.85–1.26). However, the confidence intervals for incident and persistent overweight overlapped substantially, indicating no clear evidence of a difference in the strength of these associations. These results are summarized in Table 3.

**Table 3:** Overweight status between ages 3 and 6 years

Proportions, *n *(%)						
Sex	Delivery mode, *N*		Never overweight (Non → Non)	Remitted overweight (Ove → Non)	Incident overweight (Non → Ove)	Persistent overweight (Ove → Ove)
Male	Vaginal, 24,513		21,536 (87.9%)	1,053 (4.3%)	1,389 (5.7%)	535 (2.2%)
	Cesarean, 5,563		4,777 (85.9%)	251 (4.5%)	377 (6.8%)	158 (2.8%)
Female	Vaginal, 23,456		19,954 (85.1%)	1,296 (5.5%)	1,311 (5.6%)	895 (3.8%)
	Cesarean, 5,282		4,367 (82.7%)	297 (5.6%)	367 (6.9%)	251 (4.8%)
Odds ratios						
Outcome		Sex	Crude OR	95% CI	Adjusted OR	95% CI
Incident overweight		Male	1.22	1.09–1.38	1.08	0.95–1.21
		Female	1.28	1.13–1.44	1.14	1.01–1.29
Persistent overweight		Male	1.24	0.99–1.55	1.08	0.86–1.36
		Female	1.22	1.01–1.48	1.04	0.85–1.26

Covariates: maternal pre-pregnancy BMI, maternal smoking, and maternal diabetes mellitus were included as covariates. *Non,* without overweight; *Ove,* overweight; *OR,* odds ratio; *CI,* confidence interval

### No earlier AR and limited association with post-AR weight gain in the CD group

**Fig. 1:**
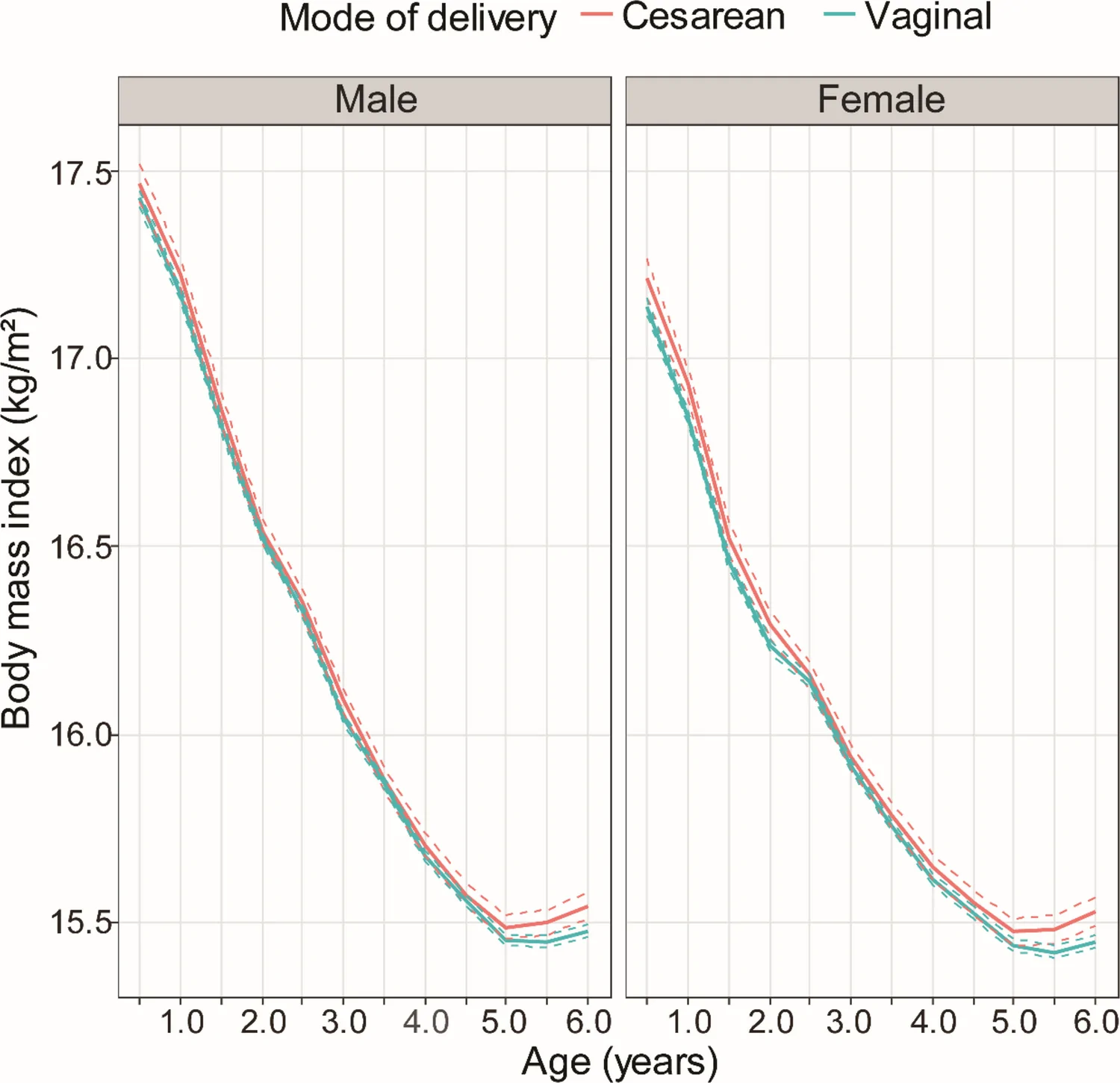
BMI trajectories from early childhood to 6 years of age according to mode of delivery, stratified by sex. The solid lines represent the mean values, and the dashed lines represent the 95% confidence intervals

To further characterize BMI development in relation to delivery mode, we examined AR timing and post-AR BMI gain. The BMI trajectories in both the CD and VD groups showed an asymmetric U-shaped pattern with a nadir at approximately 5.5 years (Fig. 1).

The analysis of AR timing revealed that early AR was identified in 40.9% of the overall cohort. Although early AR was slightly more frequent among children born by CD, no significant association was observed after adjustment (Supplementary Table 3). The BMI difference between the CD and VD groups widened during the later preschool period (Supplementary Fig. 3). Among children in whom AR was identified, the BMI slope from AR to age 6 years was greater in the CD group, with a significant difference observed among females in the crude analysis (mean difference 0.06 kg/m^2^/year, 95% CI 0.02–0.09), but not among males (0.03 kg/m^2^/year, 95% CI − 0.01–0.08). After adjustment for maternal pre-pregnancy BMI, maternal smoking, and maternal diabetes mellitus, the differences were attenuated and were no longer statistically significant in either males (adjusted difference 0.01 kg/m^2^/year, 95% CI − 0.04–0.05) or females (0.03 kg/m^2^/year, 95% CI − 0.01–0.06) (Supplementary Table 4).

## Discussion

In this large-scale prospective birth cohort study in Japan, we examined the association between CD and childhood overweight at age 6 years. Children born by CD had a higher incidence of new-onset overweight between ages 3 and 6 years among girls, whereas no significant differences were observed in AR timing or adjusted post-AR BMI gain.

Previous studies that investigated the association between CD and childhood overweight at age 6 years yielded inconsistent results; some reported positive associations and others found no significant relationship [35–37]. However, a recent meta-analysis suggests that CD is associated with a 10–20% increased risk of childhood overweight up to age 6 years [38], which supports the possibility of a modest yet meaningful association. In this study, the association remained significant after adjusting for the prespecified covariates, reinforcing the existing evidence in a Japanese population. Despite differences in BMI distribution and lifestyle between Japanese and other populations, the similar trend observed suggests potential generalizability across racial and cultural backgrounds. The overall interpretation was broadly unchanged in sensitivity analyses using alternative BMI cutoffs and classifying CD as elective or emergency.

A notable finding of this study was the modest association between CD and incident overweight between ages 3 and 6 years, which remained statistically significant after adjustment among females. However, there was no significant interaction between sex and delivery mode, providing no clear evidence that the association differed by sex. Similarly, the effect estimates and confidence intervals for incident and persistent overweight did not provide clear evidence that the strength of the association with CD differed between these trajectories. The smaller number of children with persistent overweight resulted in wider confidence intervals and lower statistical precision; therefore, the difference in statistical significance between incident and persistent overweight should be interpreted cautiously. Age 3 years marks a transitional period when infant growth stabilizes and body composition begins to track toward school-age patterns, while associations with early perinatal factors may begin to attenuate. Indeed, previous studies suggest that associations related to delivery mode weaken over time, becoming less evident by school age [39]. Similarly, some studies suggest that the association with overweight attenuates with age [40]. Thus, the observed association with incident overweight, particularly among females, may reflect modest susceptibility to weight gain during the preschool years, although our findings do not demonstrate a clear difference in the association of CD with incident versus persistent overweight.

To explore this possibility further, we analyzed AR and subsequent BMI gain. Previous studies examining the association between CD and AR have shown inconsistent results; some reported earlier AR among females born by CD [41], whereas others found no association [42]. In our cohort, we did not observe a significant association between CD and AR timing. Although post-AR BMI gain was greater among children born by CD, particularly among females in the crude analysis, the association was attenuated and no longer statistically significant after adjustment for maternal pre-pregnancy BMI, maternal smoking, and maternal diabetes mellitus. Post-AR BMI gain reflects increasing adiposity [20], and accelerated BMI gain between ages 2 and 6 years has been identified as a strong predictor of later obesity [43]. These findings provide little evidence of an association between CD and AR timing or subsequent BMI gain after adjustment.

The attenuation of the association with post-AR BMI gain after adjustment suggests that maternal and perinatal factors may contribute to the observed differences in growth trajectories. Recent Japanese cohort data also suggest that other perinatal factors, such as in vitro fertilization conception, are not necessarily associated with an increased risk of childhood obesity [44]. From a clinical perspective, children born by CD may represent a group in whom growth trajectories warrant attention, although the magnitude of the observed association was small.

This study has several limitations. First, although the adjusted models included maternal pre-pregnancy BMI, maternal smoking, and maternal diabetes mellitus as identified using the DAG, breastfeeding and early-childhood lifestyle factors, such as diet and physical activity, were unavailable or not included in the analyses. Therefore, residual confounding by these and other unmeasured factors cannot be excluded. Second, the primary analysis did not distinguish between elective and emergency CD. Although the association with childhood overweight was more evident for elective CD in the sensitivity analysis, this may reflect residual confounding by maternal or obstetric factors related to planned CD rather than an association attributable to delivery mode itself. This finding should also be interpreted cautiously because elective and emergency CD were classified indirectly based on recorded obstetric indications. Third, the study population was Japanese and the generalizability of the findings to other populations is unclear. Fourth, baseline characteristics differed between included and excluded participants, suggesting potential selection bias due to attrition. Because both delivery mode and factors associated with childhood overweight differed between the groups, the direction and magnitude of this bias are difficult to predict. Finally, because longitudinal BMI data were available only up to 6 years of age, nearly half of the participants could not be classified for AR, which commonly occurs between ages 6 and 8 years. Therefore, the observed BMI trajectories cannot be interpreted as definitive evidence that AR mediates the association between CD and childhood overweight. Longer follow-up is needed to clarify the potential role of AR in this association.

In conclusion, CD was modestly associated with childhood overweight at age 6 years. However, there was little evidence of an association between CD and AR timing or post-AR BMI gain after adjustment for maternal factors. These findings should be interpreted cautiously because residual confounding and selection bias cannot be excluded.

## Supplementary Information

Below is the link to the electronic supplementary material.

Supplementary file1 (DOCX 668 KB)

## Data Availability

Data are unsuitable for public deposition due to ethical restrictions and legal framework of Japan. It is prohibited by the Act on the Protection of Personal Information (Act No. 57 of 30 May 2003, amendment on 9 September 2015) to publicly deposit the data containing personal information. Ethical Guidelines for Medical and Health Research Involving Human Subjects enforced by the Japan Ministry of Education, Culture, Sports, Science and Technology and the Ministry of Health, Labour and Welfare also restricts the open sharing of the epidemiologic data. All inquiries about access to data should be sent to: jecs-en@nies.go.jp. The person responsible for handling enquiries sent to this e-mail address is Dr Shoji F. Nakayama, JECS Programme Office, National Institute for Environmental Studies.

## Abbreviations

aOR: Adjusted odds ratio
AR: Adiposity rebound
BMI: Body mass index
CD: Cesarean delivery
CI: Confidence interval
cOR: Crude odds ratio
IOTF: International Obesity Task Force
JECS: Japan Environment and Children’s Study
VD: Vaginal delivery

## References

[1] GBD (2024) National-level and state-level prevalence of overweight and obesity among children, adolescents, and adults in the USA, 1990–2021, and forecasts up to 2050. Lancet 404:2278–2298. 10.1016/S0140-6736(24)01548-4 PMC11694015 39551059

[2] de Onís M, Blössner M, Borghi E (2010) Global prevalence and trends of overweight and obesity among preschool children. Am J Clin Nutr 92:1257–1264. 10.3945/ajcn.2010.29786 20861173

[3] Fujiwara S, Harada K, Hagiya H et al (2024) Trends in childhood obesity in Japan: a nationwide observational study from 2012 to 2021. Clin Obes 14:e12636. 10.1111/cob.12636 38156435

[4] Spalding KL, Arner E, Westermark PO et al (2008) Dynamics of fat cell turnover in humans. Nature 453:783–787. 10.1038/nature06902 18454136

[5] Hinte LC, Castellano-Castillo D, Ghosh A et al (2024) Adipose tissue retains an epigenetic memory of obesity after weight loss. Nature 636:457–465. 10.1038/s41586-024-08165-7 PMC11634781 39558077

[6] Simmonds M, Llewellyn A, Owen CG, Woolacott N (2016) Predicting adult obesity from childhood obesity: a systematic review and meta-analysis. Obes Rev 17:95–107. 10.1111/obr.12334 26696565

[7] Stinson SE, Jonsson AE, Lund MAV et al (2021) Fasting plasma GLP-1 is associated with overweight/obesity and cardiometabolic risk factors in children and adolescents. J Clin Endocrinol Metab 106:1718–1727. 10.1210/clinem/dgab098 PMC8118577 33596309

[8] Stinson SE, Fernández de Retana Alzola I, Brünner Hovendal ED, Lund MAV, Fonvig CE, Holm LA, Jonsson AE, Frithioff-Bøjsøe C, Christiansen M, Pedersen O, Ängquist L, Sørensen TIA, Holst JJ, Hartmann B, Holm J-C, Hansen T (2024) Altered glucagon and GLP-1 responses to oral glucose in children and adolescents with obesity and insulin resistance. J Clin Endocrinol Metab 109:1590–1600. 10.1210/clinem/dgad728 PMC11099488 38087928

[9] Antoniotti V, Amore M, Caputo M et al (2023) Glucose alterations, insulin resistance, arterial hypertension, and renin are strictly associated in pediatric obesity. J Endocr Soc 7:bvad088. 10.1210/jendso/bvad088 PMC10326241 37424701

[10] Putri RR, Danielsson P, Ekström N et al (2025) Effect of pediatric obesity treatment on long-term health. JAMA Pediatr 179:302–309. 10.1001/jamapediatrics.2024.5552 PMC11877215 39836390

[11] Wang Y, Lobstein T (2006) Worldwide trends in childhood overweight and obesity. Int J Pediatr Obes 1:11–25. 10.1080/17477160600586747 17902211

[12] Woo Baidal JA, Locks LM, Cheng ER, Blake-Lamb TL, Perkins ME, Taveras EM (2016) Risk factors for childhood obesity in the first 1,000 days: a systematic review. Am J Prev Med 50:761–779. 10.1016/j.amepre.2015.11.012 26916261

[13] Monasta L, Batty GD, Cattaneo A et al (2010) Early-life determinants of overweight and obesity: a review of systematic reviews. Obes Rev 11:695–708. 10.1111/j.1467-789X.2010.00735.x 20331509

[14] Kansra AR, Lakkunarajah S, Jay MS (2021) Childhood and adolescent obesity: a review. Front Pediatr 8:581461. 10.3389/fped.2020.581461 PMC7835259 33511092

[15] Sandall J, Tribe RM, Avery L et al (2018) Short-term and long-term effects of caesarean section on the health of women and children. Lancet 392:1349–1357. 10.1016/S0140-6736(18)31930-5 30322585

[16] Li HT, Zhou YB, Liu JM (2013) The impact of cesarean section on offspring overweight and obesity: a systematic review and meta-analysis. Int J Obes (Lond) 37:893–899. 10.1038/ijo.2012.195 23207407

[17] Kuhle S, Tong OS, Woolcott CG (2015) Association between caesarean section and childhood obesity: a systematic review and meta-analysis. Obes Rev 16:295–303. 10.1111/obr.12267 25752886

[18] Terashita S, Yoshida T, Matsumura K, Hatakeyama T, Inadera H, Japan Environment and Children’s Study (JECS) Group (2023) Caesarean section and childhood obesity at age 3 years derived from the Japan Environment and Children’s Study. Sci Rep 13:6535. 10.1038/s41598-023-33653-7

[19] Rolland-Cachera MF, Deheeger M, Bellisle F, Sempé M, Guilloud-Bataille M, Patois E (1984) Adiposity rebound in children: a simple indicator for predicting obesity. Am J Clin Nutr 39:129–135. 10.1093/ajcn/39.1.129 6691287

[20] Pomi AL, Pepe G, Aversa T et al (2024) Early adiposity rebound: predictors and outcomes. Ital J Pediatr 50:98. 10.1186/s13052-024-01671-4 PMC11094876 38750561

[21] Freedman DS, Kettel Khan L, Serdula MK, Srinivasan SR, Berenson GS (2001) BMI rebound, childhood height and obesity among adults: the Bogalusa Heart Study. Int J Obes Relat Metab Disord 25:543–549. 10.1038/sj.ijo.0801581 11319660

[22] Aris IM, Rifas-Shiman SL, Li LJ et al (2019) Patterns of body mass index milestones in early life and cardiometabolic risk in early adolescence. Int J Epidemiol 48:157–167. 10.1093/ije/dyy286 PMC6380298 30624710

[23] Ohlsson C, Lorentzon M, Norjavaara E, Kindblom JM (2012) Age at adiposity rebound is associated with fat mass in young adult males-the GOOD study. PLoS ONE 7:e49404. 10.1371/journal.pone.0049404 PMC3498114 23166661

[24] Michikawa T, Nitta H, Nakayama SF et al (2018) Baseline profile of participants in the Japan Environment and Children’s Study (JECS). J Epidemiol 28:99–104. 10.2188/jea.JE20170018 PMC5792233 29093304

[25] Kawamoto T, Nitta H, Murata K et al (2014) Rationale and study design of the Japan Environment and Children’s Study (JECS). BMC Public Health 14:25. 10.1186/1471-2458-14-25 PMC3893509 24410977

[26] Gebremedhin S (2015) Multiple births in sub-Saharan Africa: epidemiology, postnatal survival, and growth pattern. Twin Res Hum Genet 18:100–107. 10.1017/thg.2014.82 25544149

[27] Cole TJ, Bellizzi MC, Flegal KM, Dietz WH (2000) Establishing a standard definition for child overweight and obesity worldwide: international survey. BMJ 320:1240–1243. 10.1136/bmj.320.7244.1240 PMC27365 10797032

[28] Kato N, Takimoto H, Sudo N (2011) The cubic functions for spline smoothed L, S and M values for BMI reference data of Japanese children. Clin Pediatr Endocrinol 20:47–49. 10.1297/cpe.20.47 PMC3687634 23926394

[29] Linares J, Corvalán C, Galleguillos B et al (2016) The effects of pre-pregnancy BMI and maternal factors on the timing of adiposity rebound in offspring. Obesity Silver Spring 24:1313–1319. 10.1002/oby.21490 27086475

[30] Textor J, van der Zander B, Gilthorpe MS et al (2016) Robust causal inference using directed acyclic graphs: the R package “dagitty.” Int J Epidemiol 45:1887–1894. 10.1093/ije/dyw341 28089956

[31] Nakamura A, Pryor L, Ballon M et al (2020) Maternal education and offspring birth weight for gestational age: the mediating effect of smoking during pregnancy. Eur J Public Health 30:1001–1006. 10.1093/eurpub/ckaa076 32529232

[32] Hartel TC, Turawa EB, Oelofse A, De Smidt JJA (2022) Effect of maternal cigarette smoking and alcohol consumption during pregnancy on birth weight and cardiometabolic risk factors in infants, children and adolescents: a systematic review protocol. BMJ Open 12:e061811. 10.1136/bmjopen-2022-061811 PMC9295650 35840294

[33] Suzuki K (2015) Longitudinal analyses of childhood growth: evidence from Project Koshu. J Epidemiol 25:2–7. 10.2188/jea.JE20140130 PMC4275431 25283310

[34] Maeda H, Hashimoto K, Iwasa H et al (2025) Association of cesarean section with asthma and atopic dermatitis in infants from the Japan Environment and Children’s Study. Sci Rep 15:39700. 10.1038/s41598-025-23252-z PMC12612077 41224837

[35] Miyayama C, Morisaki N, Ogawa K et al (2023) Evaluating the association between caesarean delivery and weight status in early childhood in a Japanese birth cohort study. Sci Rep 13:19612. 10.1038/s41598-023-45316-8 PMC10638261 37949883

[36] Portela DS, Vieira TO, Matos SM, de Oliveira NF, Vieira GO (2015) Maternal obesity, environmental factors, cesarean delivery and breastfeeding as determinants of overweight and obesity in children: results from a cohort. BMC Pregnancy Childbirth 15:94. 10.1186/s12884-015-0518-z PMC4407299 25884808

[37] Moreno-Galarraga L, Romanos Nanclares A, García-Blanco L et al (2021) Caesarean delivery is associated with an absolute increase in the prevalence of overweight in the offspring: the SENDO project. J Paediatr Child Health 57:819–825. 10.1111/jpc.15328 33426731

[38] Zhou C, Tang L, Zhou L et al (2025) Risk of early childhood overweight/obesity following cesarean section: a systematic review and meta-analysis. Int J Gynaecol Obstet 171:1092–1103. 10.1002/ijgo.70301 40521792

[39] Bråbäck L, Ekéus C, Lowe AJ, Hjern A (2013) Confounding with familial determinants affects the association between mode of delivery and childhood asthma medication—a national cohort study. Allergy Asthma Clin Immunol 9:14. 10.1186/1710-1492-9-14 PMC3643829 23590822

[40] Pei Z, Heinrich J, Fuertes E et al (2014) Cesarean delivery and risk of childhood obesity. J Pediatr 164:1068-1073.e2. 10.1016/j.jpeds.2013.12.044 24508442

[41] Zhang S, Zhou J, Yang M et al (2022) Sex-specific association between elective cesarean section and growth trajectories in preschool children: a prospective birth cohort study. Front Public Health 10:985851. 10.3389/fpubh.2022.985851 PMC9530938 36203696

[42] Baldassarre ME, Di Mauro A, Caroli M et al (2020) Premature birth is an independent risk factor for early adiposity rebound: longitudinal analysis of BMI data from birth to 7 years. Nutrients 12:3654. 10.3390/nu12123654 PMC7760942 33261215

[43] Geserick M, Vogel M, Gausche R et al (2018) Acceleration of BMI in early childhood and risk of sustained obesity. N Engl J Med 379:1303–1312. 10.1056/NEJMoa1803527 30281992

[44] Matsumoto N, Mitsui T, Kadowaki T et al (2025) In vitro fertilization and long-term child health and development: nationwide birth cohort study in Japan. Eur J Pediatr 184:24. 10.1007/s00431-024-05883-y PMC11573831 39557744

