# Supplementary material for: Cesarean delivery and overweight at 6 years of age: a prospective cohort analysis of the Japan Environment and Children’s Study
Source: Eur J Pediatr. 2026 Oct 5;185(11):803. doi: 10.1007/s00431-026-07453-w (PMC13638672; doi:10.1007/s00431-026-07453-w)
Supplement: Supplementary file 1 [file 431_2026_7453_MOESM1_ESM.docx]

Supplementary Figure 1

Flow diagram showing the recruitment process and reasons for exclusion in this study.

Supplementary Figure 2

Directed acyclic graph for the association between cesarean delivery and overweight at age 6 years.

Yellow and blue nodes indicate the exposure and outcome, respectively. Node colors indicate their causal relationship with the exposure and/or outcome. Green arrows represent causal paths, and pink arrows represent biasing paths.

Supplementary Figure 3

Differences in BMI change (ΔBMI) according to mode of delivery, stratified by sex.

The black solid line indicates the mean ΔBMI, and the shaded gray area represents the 95% confidence interval.

Supplementary Table 1

Definitions of maternal comorbidities, pregnancy complications, and neonatal complications

| Category | Conditions included |
| --- | --- |
| Maternal comorbidities | hypertension, hyperthyroidism, hypothyroidism, diabetes mellitus, autoimmune disease, heart disease, kidney disease, hepatitis, cerebral infarction, intracerebral hemorrhage, epilepsy, hematologic disease, malignancy, psychiatric disorder, neurologic disease, thrombosis, and other conditions |
| Pregnancy complications | threatened miscarriage, threatened preterm labor, premature rupture of membranes, gestational diabetes mellitus, placenta previa, non-reassuring fetal status, oligohydramnios, polyhydramnios, meconium-stained amniotic fluid, amniotic fluid embolism, placental abruption, placenta accreta, fetal growth restriction, intrauterine fetal death, hypertensive disorders of pregnancy, and other complications |
| Neonatal complications | presence/absence only; specific diagnoses were not available |

Information on maternal comorbidities, pregnancy complications, and neonatal complications was obtained from questionnaires completed by healthcare professionals. The presence or absence of each condition was determined by the attending physician.

Supplementary Table 2

Comparison of baseline characteristics between participants included in and excluded from the analysis

| Variable | Included  n=64,281 (%) | Excluded  n=28,486 (%) | SMD | P value |
| --- | --- | --- | --- | --- |
| Delivery mode |  |  | 0.04 | <0.01 |
| Vaginal delivery | 52,410 (81.5) | 22,607 (80.1) |  |  |
| Cesarean delivery | 11,871 (18.5) | 5,632 (19.9) |  |  |
| Maternal age (years) |  |  | 0.29 | <0.01 |
| <25 | 5,763 (9.0) | 5,102 (17.9) |  |  |
| 25–<30 | 18,595 (28.9) | 8,646 (30.4) |  |  |
| 30–<35 | 23,160 (36.0) | 9,060 (31.8) |  |  |
| ≥35 | 16,763 (26.1) | 5,669 (19.9) |  |  |
| Maternal BMI (kg/m²) |  |  | 0.12 | <0.01 |
| <18.5 | 10,533 (16.4) | 4,491 (15.8) |  |  |
| 18.5–<25 | 47,689 (74.2) | 20,144 (71.0) |  |  |
| ≥25 | 6,059 (9.4) | 3,727 (13.1) |  |  |
| Parity |  |  | 0.09 | <0.01 |
| Primipara | 29,313 (45.9) | 11,196 (41.5) |  |  |
| Multipara | 34,587 (54.1) | 15,755 (58.5) |  |  |
| Highest education level  (years of education) |  |  | 0.22 | <0.01 |
| <16 | 48,276 (75.7) | 22,599 (84.4) |  |  |
| ≥16 | 15,479 (24.3) | 4,187 (15.6) |  |  |
| Annual household income (million JPY) |  |  | 0.19 | <0.01 |
| <4 | 22,602 (37.6) | 11,275 (46.4) |  |  |
| 4–<6 | 20,363 (33.8) | 7,535 (31.0) |  |  |
| ≥6 | 17,197 (28.6) | 5,513 (22.7) |  |  |
| Maternal smoking |  |  | 0.31 | <0.01 |
| Never | 39,875 (62.0) | 13,043 (49.2) |  |  |
| Former | 22,306 (34.7) | 11,203 (42.3) |  |  |
| Current | 2,100 (3.3) | 2,269 (8.6) |  |  |
| Maternal alcohol intake |  |  | 0.06 | <0.01 |
| Never | 23,229 (36.2) | 9,245 (34.2) |  |  |
| Former | 35,014 (54.6) | 15,519 (57.4) |  |  |
| Current | 5,918 (9.2) | 2,257 (8.4) |  |  |
| Maternal comorbidities |  |  | 0.01 | 0.19 |
| No | 53,515 (84.8) | 23,738 (85.1) |  |  |
| Yes | 9,619 (15.2) | 4,154 (14.9) |  |  |
| Maternal diabetes |  |  | 0.01 | 0.20 |
| No | 63,600 (98.9) | 26,349 (98.8) |  |  |
| Yes | 681 (1.1) | 309 (1.2) |  |  |
| Pregnancy complications |  |  | 0.10 | <0.01 |
| No | 61,083 (95.4) | 27,535 (97.4) |  |  |
| Yes | 2,914 (4.6) | 742 (2.6) |  |  |
| Child sex |  |  | 0.00 | 0.70 |
| Male | 32,951 (51.3) | 14,637 (51.4) |  |  |
| Female | 31,330 (48.7) | 13,838 (48.6) |  |  |
| Neonatal complication |  |  | 0.01 | 0.08 |
| No | 56,390 (89.9) | 24,781 (89.5) |  |  |
| Yes | 6,327 (10.1) | 2,901 (10.5) |  |  |
| Birth height (cm) | 49.0 [48.0, 50.2] | 49.0 [47.6, 50.0] | 0.05 | <0.01 |
| Birth weight (g) | 3,028  [2,782, 3,280] | 3,024  [2,774, 3,286] | 0.02 | 0.17 |

Data are presented as median [IQR] or n (%). Percentages were calculated among participants with available data for each variable. SMD, standardized mean difference. An absolute SMD ≥0.10 was considered indicative of a potentially meaningful difference between groups. ≥16 years of education corresponds to university graduation or higher. JPY 1 million was approximately equivalent to EUR 5,556 (JPY 180/EUR, [September, 2026]).

Supplementary table 3

Occurrence of early adiposity rebound according to sex and delivery mode

| Sex | Delivery mode | N | EAR occurrence, n (%) | Crude OR (95% CI) | Adjusted OR (95% CI) |
| --- | --- | --- | --- | --- | --- |
| Overall | — | 64,281 | 26,323 (40.9) | — | — |
| Male | Vaginal | 26,854 | 10,482 (39.0) | Ref. | Ref. |
|  | Cesarean | 6,097 | 2,432 (39.9) | 1.04  (0.98–1.10) | 1.00  (0.95–1.06) |
| Female | Vaginal | 25,556 | 10,886 (42.6) | Ref. | Ref. |
|  | Cesarean | 5,774 | 2,523 (43.7) | 1.05  (0.99–1.11) | 1.01  (0.96–1.07) |

Covariates: maternal pre-pregnancy BMI, maternal smoking, and maternal diabetes mellitus were included as covariates. EAR: early adiposity rebound, OR: odds ratio, CI: confidence interval, Ref: Reference.

Supplementary Table 4

Comparison of BMI slope from AR to 6 years by delivery mode

| Sex | Delivery mode | N | Mean  (kg/m^2^/year) | Crude difference (95% CI) | Adjusted difference (95% CI) |
| --- | --- | --- | --- | --- | --- |
| Male | Vaginal | 10,482 | 0.54 | Ref. | Ref. |
|  | Cesarean | 2,432 | 0.57 | +0.03 (−0.01–0.08) | +0.01 (−0.04–0.05) |
| Female | Vaginal | 10,886 | 0.47 | Ref. | Ref. |
|  | Cesarean | 2,523 | 0.53 | +0.06 (0.02–0.09) | +0.03 (−0.01–0.06) |

Covariates: maternal pre-pregnancy BMI, maternal smoking, and maternal diabetes mellitus were included as covariates. CI: confidence interval, Ref.: Reference.
